# Oculomotor Abnormalities in Anti-Glutamic Acid Decarboxylase-Positive Stiff Person Syndrome

**DOI:** 10.3390/neurolint17110179

**Published:** 2025-11-03

**Authors:** Pavol Skacik, Jaroslav Petrisin, Kristian Sveda, Monika Turcanova-Koprusakova, Milan Grofik, Stefan Sivak, Egon Kurca

**Affiliations:** 1 Neurology Department, University Hospital Martin, Kollarova 2, 036 01 Martin, Slovakia; 2 Jessenius Faculty of Medicine Martin, Commenius University Bratislava, Mala Hora 4, 036 01 Martin, Slovakia

**Keywords:** stiff person syndrome, oculomotor dysfunction, eye movement, videonystagmography, anti-GAD

## Abstract

**Background:** Antibodies to glutamic acid decarboxylase (anti-GAD) can give rise to stiff person syndrome (SPS), an infrequent autoimmune condition of the central nervous system marked by fluctuating muscular rigidity and stimulus-evoked spasms. Disturbances in eye-movement control are rarely identified yet may provide insight into underlying neural involvement. **Methods:** Two individuals with anti-GAD-related SPS showing distinctive ocular-motor abnormalities were examined with quantitative videonystagmography, supplemented by representative video documentation. **Results:** Recordings demonstrated varied patterns of ocular-motor disturbance, including reduced smooth-pursuit accuracy, delayed saccadic initiation, dysmetria, intrusive saccades, and several nystagmus types. Partial improvement occurred after immunomodulatory therapy. **Conclusions:** These findings extend current understanding of the anti-GAD SPS phenotype and indicate that quantitative analysis of eye movements may offer a sensitive, non-invasive marker of disease activity. Larger, prospective studies are needed to clarify prevalence and responsiveness to treatment.

## 1. Background

Stiff person syndrome (SPS) represents a rare, immune-mediated disorder of the nervous system in which antibodies directed against glutamic acid decarboxylase (anti-GAD) interfere with γ-aminobutyric acid (GABA) synthesis. Loss of GABAergic inhibition produces sustained motor over-activity, and epidemiological data suggest a prevalence of roughly one to two individuals per million, most frequently in the third to fifth decades of life [1].

In clinical terms, anti-GAD–related SPS manifests through episodic rigidity and painful muscle spasms, chiefly involving the axial and proximal limb muscles. Attacks are often triggered or worsened by sudden sensory input or emotional stress. Postural deformities such as hyperlordosis, impaired gait, and co-existing psychological disturbance—especially anxiety or depression—are common accompaniments. Without timely intervention, progressive loss of motor function and increasing disability are likely [2].

Diagnosis is grounded in clinical observation but strengthened by ancillary testing. Electromyography typically reveals continuous motor-unit discharges, and detection of anti-GAD antibodies in serum or cerebrospinal fluid, together with improvement following GABA-enhancing medication such as benzodiazepines, supports the diagnosis. Classification into definite or probable SPS follows internationally accepted criteria [3,4]. Autoimmune clustering is frequent: associations are documented with type 1 diabetes, autoimmune thyroid disease, coeliac disease, pernicious anaemia, and myasthenia gravis, together with a recognised linkage to HLA class II alleles [4].

Disturbances in ocular motility have occasionally been observed in SPS yet are rarely identified during routine assessment [5,6]. Because anti-GAD antibodies may compromise GABA-dependent pathways within the brainstem and cerebellum—regions essential for the coordination of eye movements—systematic neuro-ophthalmological evaluation could provide a sensitive, non-invasive means of detecting central nervous system involvement [7].

The present study details two representative patients with anti-GAD-associated SPS who exhibited distinctive eye-movement patterns documented by videonystagmography. By outlining these observations, we aim to broaden clinical awareness of ocular-motor dysfunction within the SPS spectrum and to emphasise its potential diagnostic and monitoring value.

## 2. Case Presentation 1

History: A 40-year-old man presented with a two-year history of progressive neuromuscular symptoms, including intolerance to physical activity and sensory stimuli that provoked episodic painful muscle cramps and fatigue. These symptoms most prominently affected the facial region, abdominal musculature, and lower limbs.

Clinical findings: Neurological examination revealed asymmetric blepharospasm and transient right-sided ptosis, both consistently elicited during oculomotor testing, particularly with upward gaze. No nystagmus or signs of bulbar involvement were observed. During assessment of facial muscle innervation, repeated movements (such as smiling or baring the teeth) elicited bradykinesia and a progressive reduction in movement amplitude. Examination of the limbs demonstrated normal muscle tone and no evidence of paresis. Pyramidal signs were present, including bilateral Babinski responses and hyperreflexia in the lower limbs. Bradykinesia was again observed during repetitive motor tasks, including finger tapping and facial movements. Psychological assessment identified comorbid anxiety and depression.

Diagnosis: Extensive diagnostic evaluation—including serological testing, cerebrospinal fluid analysis, electrophysiological studies, and neuroimaging—was performed. Brain and spinal imaging were unremarkable. Needle electromyography demonstrated continuous spontaneous motor unit activity potentials. Paraneoplastic screening was negative. Elevated anti-GAD antibody index was detected by ELISA (antibody index 25.0; reference range 0.00–1.00). Based on these findings, together with the clinical features, a diagnosis of definite anti-GAD-associated SPS was established.

**Figure 1:**
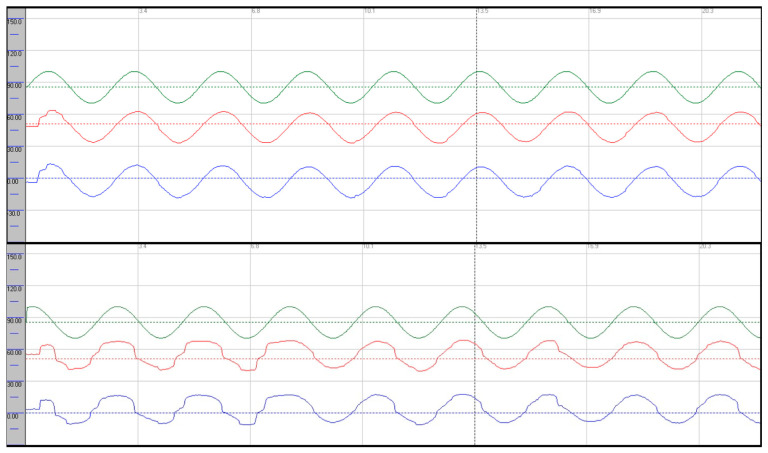
Smooth pursuit recordings in the horizontal (**upper trace**) and vertical (**lower trace**) planes, demonstrating a predominant vertical pursuit deficit. Stimulation parameters: amplitude 30°, frequency 0.4 Hz. Average gains—horizontal: right eye 0.85 (rightward), 0.87 (leftward); left eye 0.85 (rightward), 0.87 (leftward). Vertical: right eye 0.66 (upward), 0.56 (downward); left eye 0.66 (upward), 0.56 (downward). Green: target; red: right eye; blue: left eye.

**Figure 2:**
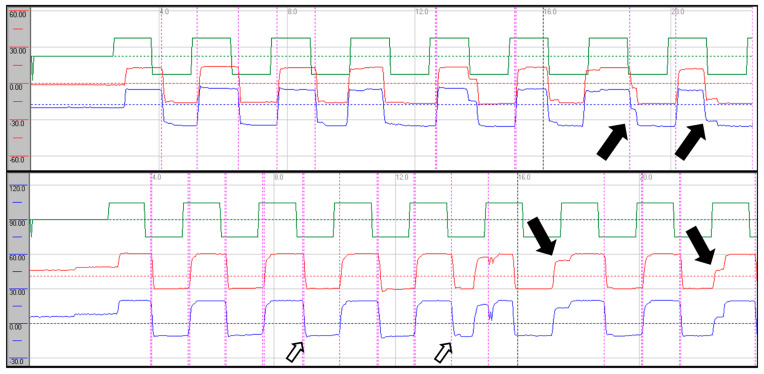
Saccadic recordings in the horizontal (**upper trace**) and vertical (**lower trace**) planes. Repetitive stimulation revealed horizontal hypometria (greater to the left) and upward hypometria (black arrows). Downward saccades show slight hypermetria (black hollow arrows). Stimulation parameters: amplitude 30°, frequency 0.4 Hz. Green: target; red: right eye; blue: left eye.

Oculomotor assessment and videonystagmography: Oculomotor testing was performed using videonystagmography (SYNAPSYS VNG Ulmer) with both monocular and binocular infrared cameras. Primary gaze was stable in all directions, with no evidence of spontaneous or gaze-evoked nystagmus, and without saccadic intrusions. Smooth pursuit was mildly impaired, particularly in the vertical plane, with reduced average values most evident in downward tracking (see Figure 1). Evaluation of saccadic eye movements demonstrated prolonged latencies in all directions. Repetitive stimulation led to the occurrence of saccadic hypometria, and slight hypermetria of downward saccades was also observed (see Figure 2). During gaze-evoked testing, upward gaze consistently provoked asymmetric blepharospasm and transient right-sided ptosis, accompanied by delayed relaxation of the orbicularis oculi muscles during eye closure and reopening (Supplementary Video S1).

Treatment. The patient was started on immunomodulatory therapy consisting of five sessions of plasmapheresis (5000 mL per session). Adjunctive treatment with muscle relaxants, physiotherapy, and neuropsychological intervention was initiated. This combination yielded clinical improvement.

Follow-up VNG showed stable smooth pursuit in the horizontal plane with predominant vertical dysfunction; there was no improvement in gain values in any direction, and vertical pursuit was slightly more impaired, particularly during downward tracking. Upward gaze continued to provoke blepharospasm-like eyelid phenomena (Supplementary Video S1). Saccadic latencies were unchanged, hypometria with repetitive stimulation was no longer observed, and vertical saccades continued to exhibit dysmetria as previously described. The eyelid phenomena persisted. Written informed consent was obtained from the patient.

## 3. Case Presentation 2

History. A 44-year-old woman presented with a four-month history of progressive cramps and muscle spasms, predominantly affecting the lower limbs. These episodes were triggered by emotional stimuli, postural changes, and sensory inputs such as tactile sensations and sounds. Between episodes, she developed increasing muscle stiffness that eventually rendered her immobile.

Clinical findings. Cranial nerve assessment revealed subtle smooth pursuit abnormalities, saccadic dysfunction, and saccadic intrusions. There was no evidence of facial nerve or bulbar involvement. Examination of the limbs showed hypertonia in all extremities with heightened reflexes and positive pyramidal signs in the lower limbs. Gait was severely impaired, displaying a spastic and occasionally magnetic pattern, with muscle spasms in the lower limbs triggered by sensory stimuli. Psychological assessment demonstrated severe anxiety and reactive depression.

Diagnosis. Extensive diagnostic evaluation, including serological testing, cerebrospinal fluid analysis, electrophysiological studies, and neuroimaging, was undertaken. Brain and spinal imaging were unremarkable. Nerve conduction studies were normal. Needle electromyography demonstrated continuous spontaneous motor unit activity potentials. Paraneoplastic screening was negative. Elevated anti-GAD antibody index was detected by ELISA (antibody index > 400.0; reference range 0.00–1.00). Based on these findings, together with the clinical features, a diagnosis of definite anti-GAD-associated SPS was established.

**Figure 3:**
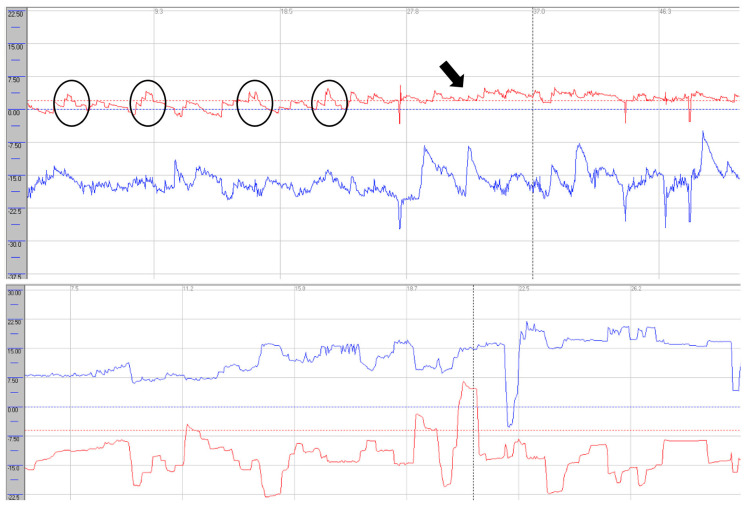
Videonystagmography during primary gaze. Horizontal traces (red) show paroxysmal saccadic intrusions (black circles) with superimposed right-beating nystagmus (black arrows). Vertical traces (blue) demonstrate continuous, arrhythmic saccadic intrusions (**upper trace**); square-wave jerks were also present (**lower trace**). Red line: horizontal eye movements; blue line: vertical eye movements.

**Figure 4:**
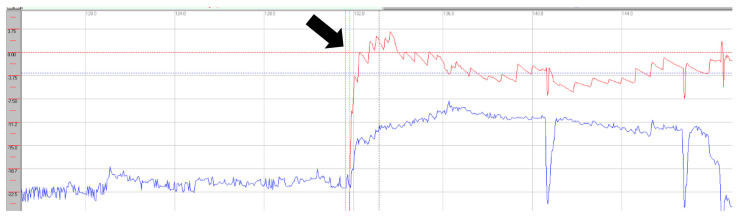
Videonystagmography during rightward gaze showing gaze-evoked right-beating horizontal nystagmus (black arrow). Red line: horizontal eye movements; blue line: vertical eye movements.

Oculomotor assessment and videonystagmography. Oculomotor testing was performed using videonystagmography (SYNAPSYS VNG Ulmer) with both monocular and binocular infrared cameras. In the primary gaze, paroxysmal saccadic intrusions were observed in the horizontal plane, together with horizontal nystagmus, as well as persistent vertical saccadic intrusions with an irregular, arrhythmic pattern (Supplementary Video S2). These phenomena were often superimposed, and square-wave jerks were also identified (see Figure 3 and Figure 4). Rightward gaze testing revealed gaze-evoked horizontal nystagmus (Supplementary Video S3). During the return to the primary position, we observed a form of rebound nystagmus (Supplementary Video S4).

**Figure 5:**
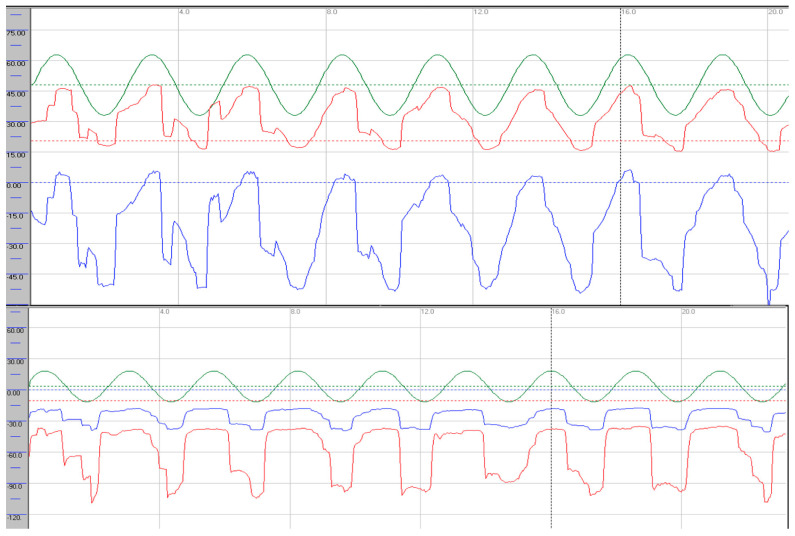
Smooth pursuit recordings in the horizontal (**upper trace**) and vertical (**lower trace**) planes, demonstrating severe pursuit deficits. Stimulation parameters: amplitude 30°, frequency 0.4 Hz. Average gains—horizontal: right eye 0.52 (rightward), 0.41 (leftward); left eye 0.52 (rightward), 0.41 (leftward). Vertical: right eye 0.48 (upward), 0.36 (downward); left eye 0.48 (upward), 0.36 (downward). Green: target; red: right eye; blue: left eye.

Smooth pursuit was severely impaired, most pronounced in the vertical plane, with reduced tracking accuracy and frequent catch-up saccades (see Figure 5).

**Figure 6:**
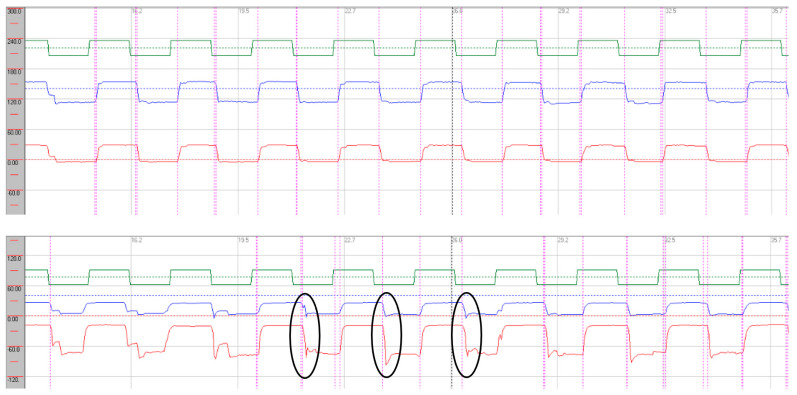
Saccadic recordings in the horizontal (**upper trace**) and vertical (**lower trace**) planes. Downward saccades demonstrate hypermetria (black circles). Stimulation parameters: amplitude 30°, frequency 0.4 Hz. Green: target; red: right eye; blue: left eye.

Horizontal saccades demonstrated normal latency without evidence of dysmetria. During vertical gaze testing, saccadic analysis revealed hypermetria of downward saccades (see Figure 6).

Treatment: Immunotherapy was initiated with corticosteroids followed by plasmapheresis. Symptomatic treatment with a combination of benzodiazepines and neuroleptics was administered to relieve muscle stiffness and cramps. Subsequently, maintenance oral prednisone was introduced, with a favourable clinical effect. On follow up, oculomotor function showed improvement, with a reduction in gaze-holding deficits; however, gaze-evoked nystagmus persisted. Smooth pursuit in the horizontal plane demonstrated improved average gain values, and the previously observed hypermetria of downward saccades had resolved. Written and informed consent was obtained from the patient.

## 4. Discussion

Anti-GAD-positive neurological disorders present with a broad phenotypical spectrum. The main clinical manifestations include stiff person syndrome (SPS), cerebellar ataxia, autoimmune epilepsy, limbic encephalitis, progressive encephalomyelitis with rigidity and myoclonus, nystagmus, and other abnormal eye movements. These syndromes may occur in isolation or with significant overlap [8].

A wide range of oculomotor abnormalities in anti-GAD SPS has been reported in the literature [7]. However, their recognition in clinical practice remains substantially underdiagnosed and underappreciated [4].

Oculomotor disturbances in anti-GAD SPS span several functional domains—those that stabilise vision on a target of interest and those that enable gaze shifts to new targets. They may range from non-specific findings to isolated nystagmus and/or oculomotor dysfunction of varying severity. Other abnormalities have also been described, including voluntary gaze provoking upper-quadrant facial hyperkinesias, conjugate eye deviations, and abduction paresis mimicking internuclear ophthalmoplegia. These disturbances are frequently exacerbated by fatigue or sensory stimuli [9,10,11]. Overlapping features with other immune-mediated neurological disorders, such as myasthenia gravis, have also been reported [6].

Central forms of nystagmus syndromes have been observed in the horizontal plane, such as gaze-evoked nystagmus, as well as in vertical forms, including downbeat nystagmus. These findings indicate dysfunction of the brainstem and cerebellar gaze integrator circuits, such as the nucleus prepositus hypoglossi and the cerebellar flocculus, both of which rely heavily on GABAergic modulation. In downbeat nystagmus syndrome, vertical vestibulo-ocular reflexes depend on a balanced interaction of the semicircular canals, with the cerebellar flocculus selectively inhibiting anterior canal pathways [12]. In case presentation 2, we observed a form of rebound nystagmus, a recognised sign of dysfunction of the neural integrator for gaze holding within brainstem–cerebellar circuits [13].

Smooth pursuit dysfunction was observed in both patients and was more pronounced in the vertical plane, with slightly greater impairment during downward gaze. The severity of smooth pursuit deficits ranged from mild in case 1 to severe disruption in case 2. Some abnormalities, particularly mild pursuit deficits, may also occur in healthy individuals and should be interpreted carefully. Moreover, differences between horizontal and vertical pursuit are well recognised, with vertical pursuit generally being less accurate, and asymmetries between right–left and up–down movements may also occur [14]. Shemesh et al. demonstrated in animal models that bilateral floccular lesions predominantly impair downward pursuit more than upward pursuit, possibly due to asymmetry in the vertical gaze-velocity Purkinje cells of the flocculus [15]. This up–down asymmetry was evident in both of our cases, with deficits most pronounced during downward pursuit, as reflected by average gain values. An additional finding was deterioration of smooth pursuit with repeated stimulation, observed in case 1, suggesting oculomotor intolerance to repetitive activation.

Saccadic abnormalities were also frequent. Both cases demonstrated prolonged latencies, more pronounced in the vertical plane and particularly during downward gaze. Normal values are approximately 200 ms [16]. In vertical downward gaze, we observed slight hypermetria of saccades in both patients, while case 1 also exhibited hypometric saccades in upward gaze. These alterations in latency and dysmetria may be attributed to dysfunction of the rostral interstitial nucleus of the medial longitudinal fasciculus (riMLF), the cerebellar vermis, and involvement of the fastigial nucleus. The presence of saccadic intrusions, opsoclonus, or flutter further supports widespread central involvement, consistent with dysfunction of omnipause neurons within the brainstem nuclei [17]. In case presentation 2, we observed a mixture of intrusion types—both with and without an intersaccadic interval—indicating that these abnormalities may overlap with each other and with other oculomotor syndromes, such as nystagmus. In this case, intrusions occurred in the horizontal plane in a paroxysmal fashion and were almost continuous in the vertical plane [18].

Oculomotor system abnormalities therefore represent additional signs in patients with anti-GAD SPS, or may reflect overlap with oculomotor dysfunction and nystagmus, which has been defined as a distinct clinical entity. Given the extensive network of oculomotor structures and functions, such abnormalities may be even more frequent than previously recognised, with some studies reporting their presence in up to 23% of patients [19]. These manifestations may often be subtle or subclinical and may only be detected using Frenzel goggles or videonystagmography. These cases expand the recognised clinical spectrum of anti-GAD-associated SPS.

Autoimmune and immune-mediated mimics of anti-GAD-associated SPS with overlapping oculomotor signs span the broader spectrum of autoimmune cerebellar ataxias (e.g., gluten ataxia, post-infectious forms, primary autoimmune cerebellar ataxia) as well as Miller–Fisher syndrome, opsoclonus–myoclonus syndrome, and paraneoplastic cerebellar degeneration [20]. The differential further extends to selected movement disorders, genetic ataxias, and infectious and metabolic aetiologies [21]; among others, ocular myasthenia should also be considered [22]. In practice, differentiation begins with careful phenomenological analysis of eye-movement abnormalities in the context of associated neurological signs: downbeat nystagmus points to cerebellar syndromes [23]; fatigable ocular motility supports myasthenia [24]; florid opsoclonus–myoclonus commonly suggests a paraneoplastic process [25]; and severe supranuclear ophthalmoplegia may indicate parkinsonian syndrome, particularly progressive supranuclear palsy (PSP) [26]. As oculomotor examination alone seldom achieves high aetiological specificity, ancillary testing—including electrophysiology, targeted antibody panels, cerebrospinal fluid analysis, neuroimaging, and tumour screening—is often warranted.

In Case 1, immunomodulatory therapy resulted in clinical improvement but showed only partial improvement on saccadic testing. In Case 2, immunomodulatory therapy led to clear improvement in oculomotor function, especially in smooth pursuit and vertical saccades, with a reduction in saccadic intrusions in the primary gaze. The beneficial effects of immunomodulation on oculomotor function have been reported with IVIG [27] and with other agents such as rituximab [28]. Improvement following immunomodulation raises the possibility of using oculomotor parameters as sensitive markers of therapeutic response, although causality cannot be established from two cases. Prospective studies with systematic evaluation of oculomotor profiles and treatment responses will be required.

## 5. Conclusions

Oculomotor abnormalities may represent clinically relevant, non-invasive markers of central oculomotor involvement in SPS. Taken together, these findings expand the recognised clinical phenotype of anti-GAD SPS. It is important to note that the observations presented in this paper are illustrative and hypothesis-generating rather than generalisable. They underscore the need for systematic, prospective studies employing quantitative oculomotor assessment, to clarify the prevalence, diagnostic significance, and therapeutic responsiveness of these abnormalities.

## Data Availability

The datasets generated and/or analysed during the current study are available from the corresponding author on request.

## Supplementary Materials

The following supporting information can be downloaded at: https://www.mdpi.com/article/10.3390/neurolint17110179/s1, Video S1: Oculomotor eyelid phenomena on upward gaze with asymmetric contraction of the right orbicularis oculi and delayed relaxation, causing impaired vertical eye tracking on VNG with infrared binocular goggles; Video S2: Videonystagmography during primary gaze showing saccadic intrusions in both the horizontal and vertical planes; Video S3: Videonystagmography demonstrating gaze-evoked nystagmus; Video S4: Videonystagmography demonstrating rebound nystagmus on return to primary position.
